# 2-Amino­pyridin-3-ol

**DOI:** 10.1107/S1600536811034775

**Published:** 2011-08-27

**Authors:** Richard Betz, Thomas Gerber, Eric Hosten, Henk Schalekamp

**Affiliations:** a Nelson Mandela Metropolitan University, Summerstrand Campus, Department of Chemistry, University Way, Summerstrand, PO Box 77000, Port Elizabeth 6031, South Africa

## Abstract

The molecule of the title pyridine derivative, C_5_H_6_N_2_O, shows approximate *C*_*s*_ symmetry. Intra­cyclic angles cover the range 118.34 (10)–123.11 (10)°. In the crystal, O—H⋯N, N—H⋯O and N—H⋯N hydrogen bonds connect the mol­ecules into double layers perpendicular to the *a* axis. The shortest centroid–centroid distance between two π-systems is 3.8887 (7) Å.

## Related literature

For the crystal structure of 2,3-diamino­pyridine, see: Betz *et al.* (2011 ▶). For graph-set analysis of hydrogen bonds, see: Etter *et al.* (1990 ▶); Bernstein *et al.* (1995 ▶). For general information about the chelate effect in coordination chemistry, see: Gade (1998 ▶).
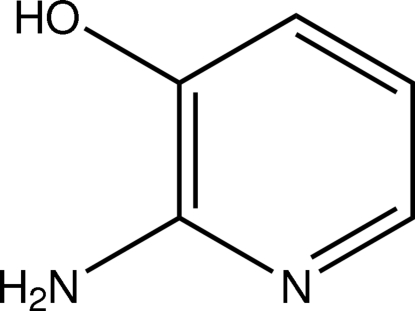


## Experimental

### 

#### Crystal data

C_5_H_6_N_2_O
*M* *_r_* = 110.12
Monoclinic, 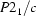

*a* = 12.5310 (6) Å
*b* = 3.8887 (2) Å
*c* = 11.6042 (5) Å
β = 113.139 (2)°
*V* = 519.98 (4) Å^3^
*Z* = 4
Mo *K*α radiation
μ = 0.10 mm^−1^
*T* = 200 K
0.29 × 0.25 × 0.13 mm

#### Data collection

Bruker APEXII CCD diffractometer
4820 measured reflections
1289 independent reflections
1008 reflections with *I* > 2σ(*I*)
*R*_int_ = 0.031

#### Refinement

*R*[*F* ^2^ > 2σ(*F* ^2^)] = 0.036
*wR*(*F* ^2^) = 0.117
*S* = 1.13
1289 reflections
81 parameters
H atoms treated by a mixture of independent and constrained refinement
Δρ_max_ = 0.29 e Å^−3^
Δρ_min_ = −0.22 e Å^−3^

### 

Data collection: *APEX2* (Bruker, 2010 ▶); cell refinement: *SAINT* (Bruker, 2010 ▶); data reduction: *SAINT*; program(s) used to solve structure: *SHELXS97* (Sheldrick, 2008 ▶); program(s) used to refine structure: *SHELXL97* (Sheldrick, 2008 ▶); molecular graphics: *ORTEP-3* (Farrugia, 1997 ▶) and *Mercury* (Macrae *et al.*, 2008 ▶); software used to prepare material for publication: *SHELXL97* and *PLATON* (Spek, 2009 ▶).

## Supplementary Material

Crystal structure: contains datablock(s) I, global. DOI: 10.1107/S1600536811034775/bh2372sup1.cif

Supplementary material file. DOI: 10.1107/S1600536811034775/bh2372Isup2.cdx

Structure factors: contains datablock(s) I. DOI: 10.1107/S1600536811034775/bh2372Isup3.hkl

Supplementary material file. DOI: 10.1107/S1600536811034775/bh2372Isup4.cml

Additional supplementary materials:  crystallographic information; 3D view; checkCIF report

## Figures and Tables

**Table 1:** Hydrogen-bond geometry (Å, °)

*D*—H⋯*A*	*D*—H	H⋯*A*	*D*⋯*A*	*D*—H⋯*A*
O1—H1⋯N1^i^	0.82	1.85	2.6639 (12)	172
N2—H71⋯O1^ii^	0.870 (17)	2.276 (17)	3.0184 (13)	143.2 (12)
N2—H72⋯N2^iii^	0.895 (18)	2.358 (17)	3.1249 (15)	143.8 (15)

Symmetry codes: (i) 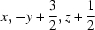; (ii) 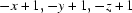; (iii) 
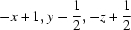
.

**Fig. 1.**
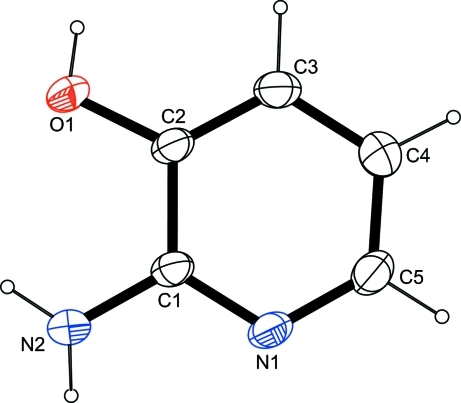
The molecular structure of the title compound, with atom labels and anisotropic displacement ellipsoids (drawn at 50% probability level).

**Fig. 2.**
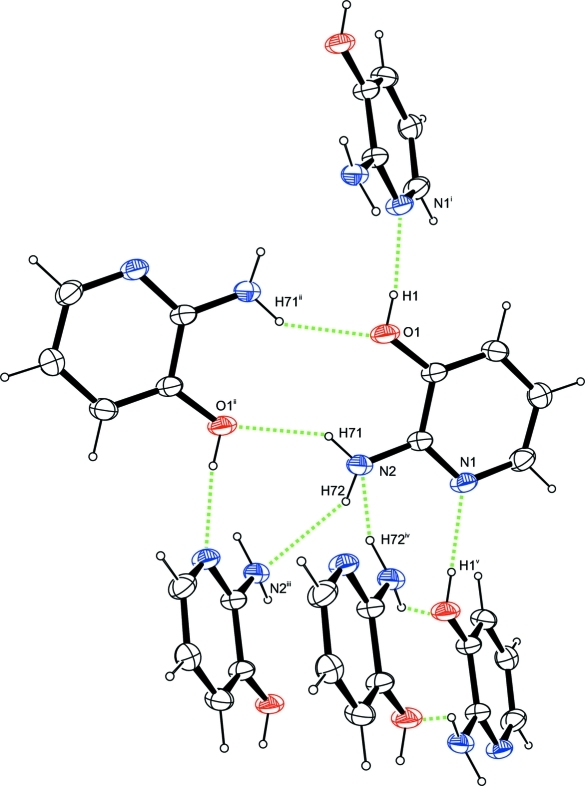
Intermolecular contacts, viewed approximately along [-1 - 1 -1]. Symmetry operators: ix, -y + 3/2, z + 1/2; ii -x + 1, -y + 1, -z + 1; iii -x + 1, y - 1/2, -z + 1/2; vi -x + 1, y + 1/2, -z + 1/2; vx, -y + 3/2, z - 1/2.

**Fig. 3.**
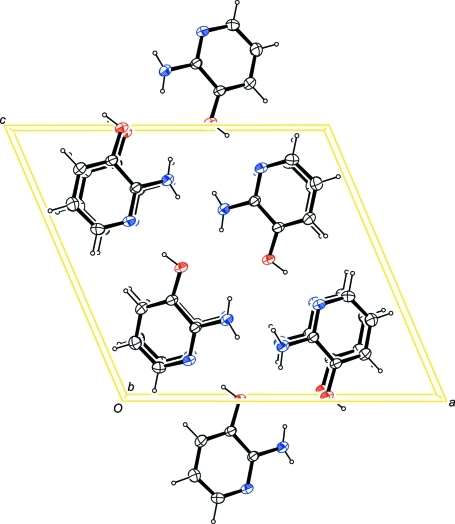
Molecular packing of the title compound, viewed along [0 1 0] (anisotropic displacement ellipsoids drawn at 50% probability level).

## supplementary crystallographic information

### Comment

Chelate ligands have found widespread use in coordination chemistry due to the enhanced thermodynamic stability of resultant metal complexes in relation to coordination compounds exclusively applying comparable monodentate ligands (Gade, 1998). Combining different donor atoms, a molecular set-up to accommodate a large variety of metal centers of variable Lewis acidity is at hand. In this aspect, the title compound seemed interesting due to its use as strictly neutral or – depending on the pH value – as anionic or cationic ligand. Furthermore, thanks to the presence of three possible donor atoms, the title compound might serve as a building block in the formation of metal-organic framework structures. At the beginning of a more comprehensive study to elucidate the formation of coordination polymers featuring mixed *N*,*O* ligands, we determined the structure of the title compound to enable comparative studies of metrical parameters in envisioned reaction products. Information about the molecular and crystal structure of 2,3-diaminopyridine is apparent in the literature (Betz *et al.*, 2011).

Intracyclic angles range from 118.34 (10) ° to 123.11 (10) ° with the smallest angle found on the carbon atom bearing the hydroxyl group and the largest angle found on the unsubstituted carbon atom in *ortho* position to the intracyclic N atom. The molecule is essentially planar (r.m.s. of all fitted non-hydrogen atoms = 0.0092 Å). The amino group is not planar, the least-squares planes defined by the atoms of the heterocycle on the one hand and the atoms of the NH_2_ group on the other hand group enclose an angle of 30.73(1.69) ° (Fig. 1).

The crystal structure of the title compound is marked by hydrogen bonds (Fig. 2). While the hydroxyl group forms a hydrogen bond to the intracyclic N atom (and its O atom acts as acceptor for one of the NH_2_ supported hydrogen bonds), there is also a cooperative hydrogen bonding system of the NH_2_···NH_2_-type. In terms of graph-set analysis (Etter *et al.*, 1990; Bernstein *et al.*, 1995), the descriptor for these interactions on the unitary level is *C*^1^_1_(2)*C*^1^_1_(5)*C*^1^_1_(5). In total, the molecules are connected to double layers perpendicular to the crystallographic *a* axis. The shortest intercentroid distance between two π-systems was measured at 3.8887 (7) Å.

The packing of the title compound in the crystal is shown in Figure 3.

### Experimental

The compound was obtained commercially (Aldrich). Crystals suitable for the X-ray diffraction study were taken directly from the provided compound.

### Refinement

Carbon-bound H atoms were placed in calculated positions (C—H 0.95 Å) and were included in the refinement in the riding model approximation, with *U*(H) set to 1.2*U*_eq_(C). The H atom of the hydroxyl group was placed in a calculated position (O—H 0.82 Å) and was included in the refinement in the riding model approximation, with *U*(H) set to 1.5*U*_eq_(O). Both nitrogen-bound H atoms were located on a difference Fourier map and refined freely.

### Crystal data

**Table:**

C_5_H_6_N_2_O	*F*(000) = 232
*M**_r_* = 110.12	*D*_x_ = 1.407 Mg m^−^^3^
Monoclinic, *P*2_1_/*c*	Mo *K*α radiation, λ = 0.71073 Å
Hall symbol: -P 2ybc	Cell parameters from 2447 reflections
*a* = 12.5310 (6) Å	θ = 3.5–28.1°
*b* = 3.8887 (2) Å	µ = 0.10 mm^−^^1^
*c* = 11.6042 (5) Å	*T* = 200 K
β = 113.139 (2)°	Block, brown
*V* = 519.98 (4) Å^3^	0.29 × 0.25 × 0.13 mm
*Z* = 4	

### Data collection

**Table:**

Bruker APEXII CCD diffractometer	1008 reflections with *I* > 2σ(*I*)
Radiation source: fine-focus sealed tube	*R*_int_ = 0.031
graphite	θ_max_ = 28.3°, θ_min_ = 1.8°
φ and ω scans	*h* = −15→16
4820 measured reflections	*k* = −5→3
1289 independent reflections	*l* = −15→15

### Refinement

**Table:**

Refinement on *F*^2^	Primary atom site location: structure-invariant direct methods
Least-squares matrix: full	Secondary atom site location: difference Fourier map
*R*[*F*^2^ > 2σ(*F*^2^)] = 0.036	Hydrogen site location: inferred from neighbouring sites
*wR*(*F*^2^) = 0.117	H atoms treated by a mixture of independent and constrained refinement
*S* = 1.13	*w* = 1/[σ^2^(*F*_o_^2^) + (0.0669*P*)^2^ + 0.0455*P*] where *P* = (*F*_o_^2^ + 2*F*_c_^2^)/3
1289 reflections	(Δ/σ)_max_ < 0.001
81 parameters	Δρ_max_ = 0.29 e Å^−^^3^
0 restraints	Δρ_min_ = −0.22 e Å^−^^3^
0 constraints	

### Fractional atomic coordinates and isotropic or equivalent isotropic displacement parameters (Å^2^)

**Table:**

	*x*	*y*	*z*	*U*_iso_*/*U*_eq_	
O1	0.36008 (7)	0.7180 (3)	0.48635 (7)	0.0334 (3)	
H1	0.3249	0.7262	0.5328	0.050*	
N1	0.26183 (8)	0.7983 (3)	0.15240 (8)	0.0262 (3)	
N2	0.43131 (8)	0.5713 (3)	0.30122 (10)	0.0290 (3)	
H71	0.4673 (14)	0.495 (4)	0.3773 (16)	0.037 (4)*	
H72	0.4404 (14)	0.444 (5)	0.2414 (17)	0.051 (5)*	
C1	0.32537 (9)	0.7224 (3)	0.27173 (10)	0.0223 (3)	
C2	0.28799 (9)	0.8073 (3)	0.36913 (10)	0.0233 (3)	
C3	0.18289 (10)	0.9681 (3)	0.33747 (11)	0.0273 (3)	
H3	0.1558	1.0290	0.4006	0.033*	
C4	0.11580 (10)	1.0419 (3)	0.21157 (11)	0.0302 (3)	
H4	0.0424	1.1509	0.1877	0.036*	
C5	0.15829 (10)	0.9537 (3)	0.12361 (10)	0.0300 (3)	
H5	0.1127	1.0043	0.0382	0.036*	

### Atomic displacement parameters (Å^2^)

**Table:**

	*U*^11^	*U*^22^	*U*^33^	*U*^12^	*U*^13^	*U*^23^
O1	0.0280 (5)	0.0579 (6)	0.0161 (4)	0.0062 (4)	0.0109 (4)	0.0035 (4)
N1	0.0277 (5)	0.0355 (6)	0.0170 (5)	−0.0017 (4)	0.0103 (4)	0.0006 (4)
N2	0.0275 (5)	0.0420 (7)	0.0207 (5)	0.0060 (4)	0.0129 (4)	0.0020 (4)
C1	0.0237 (6)	0.0271 (6)	0.0182 (5)	−0.0031 (4)	0.0106 (4)	−0.0002 (4)
C2	0.0246 (6)	0.0300 (6)	0.0167 (5)	−0.0025 (4)	0.0096 (4)	−0.0004 (4)
C3	0.0284 (6)	0.0337 (7)	0.0235 (6)	−0.0001 (5)	0.0143 (5)	−0.0026 (5)
C4	0.0251 (6)	0.0360 (7)	0.0297 (6)	0.0045 (5)	0.0109 (5)	0.0028 (5)
C5	0.0280 (6)	0.0399 (7)	0.0197 (5)	0.0003 (5)	0.0069 (4)	0.0047 (5)

### Geometric parameters (Å, °)

**Table:**

O1—C2	1.3496 (13)	C1—C2	1.4219 (14)
O1—H1	0.8200	C2—C3	1.3712 (16)
N1—C1	1.3312 (14)	C3—C4	1.3994 (16)
N1—C5	1.3490 (15)	C3—H3	0.9500
N2—C1	1.3667 (14)	C4—C5	1.3675 (16)
N2—H71	0.870 (17)	C4—H4	0.9500
N2—H72	0.895 (18)	C5—H5	0.9500
			
C2—O1—H1	109.5	C3—C2—C1	118.34 (10)
C1—N1—C5	118.74 (9)	C2—C3—C4	119.53 (10)
C1—N2—H71	117.7 (9)	C2—C3—H3	120.2
C1—N2—H72	116.8 (11)	C4—C3—H3	120.2
H71—N2—H72	115.2 (15)	C5—C4—C3	118.52 (11)
N1—C1—N2	118.74 (9)	C5—C4—H4	120.7
N1—C1—C2	121.74 (10)	C3—C4—H4	120.7
N2—C1—C2	119.46 (10)	N1—C5—C4	123.11 (10)
O1—C2—C3	125.40 (9)	N1—C5—H5	118.4
O1—C2—C1	116.25 (10)	C4—C5—H5	118.4
			
C5—N1—C1—N2	−178.69 (10)	O1—C2—C3—C4	−179.09 (11)
C5—N1—C1—C2	−1.36 (18)	C1—C2—C3—C4	0.32 (18)
N1—C1—C2—O1	−179.79 (10)	C2—C3—C4—C5	−0.72 (19)
N2—C1—C2—O1	−2.49 (17)	C1—N1—C5—C4	0.94 (19)
N1—C1—C2—C3	0.74 (18)	C3—C4—C5—N1	0.1 (2)
N2—C1—C2—C3	178.05 (11)		

### Hydrogen-bond geometry (Å, °)

**Table:**

*D*—H···*A*	*D*—H	H···*A*	*D*···*A*	*D*—H···*A*
O1—H1···N1^i^	0.82	1.85	2.6639 (12)	172.
N2—H71···O1^ii^	0.870 (17)	2.276 (17)	3.0184 (13)	143.2 (12)
N2—H72···N2^iii^	0.895 (18)	2.358 (17)	3.1249 (15)	143.8 (15)

Symmetry codes: (i) *x*, −*y*+3/2, *z*+1/2; (ii) −*x*+1, −*y*+1, −*z*+1; (iii) −*x*+1, *y*−1/2, −*z*+1/2.
